# Clinical potential and challenges of spatially profiling tumor-infiltrating lymphocytes in early-stage breast cancer

**DOI:** 10.1371/journal.pmed.1004979

**Published:** 2026-03-10

**Authors:** David B. Page, Michael Simanonok, Douglas A. Hanes, Alan Su

**Affiliations:** 1 Department of Medicine, Division of Medical Oncology, Providence Cancer Institute, Portland, Oregon, United States of America; 2 Earle A. Chiles Research Institute, Portland, Oregon, United States of America,; 3 Center for Cardiovascular Analytics, Research and Data Science (CARDS), Providence Heart Institute, Providence Research Network, Portland, Oregon

## Abstract

In this Perspective, David Page and colleagues discuss how spatially profiling immune-tumor cell interactions using multispectral immunofluorescence analyses holds promise as a biomarker to predict outcomes in early-stage breast cancer.

## Teaser

Digitally automated spatial profiling of immune-tumor cell interactions using multispectral immunofluorescence holds promise as a biomarker to predict outcomes in early-stage breast cancer, but prospective validation and harmonization with existing biomarkers is necessary before clinical adoption.

Immune checkpoint inhibitors (ICIs), including pembrolizumab and durvalumab, are a type of cancer immunotherapy that promotes anti-tumor immune responses by blocking suppressive receptor–ligand interactions, such as programmed death 1 (PD-1) and its ligand (PD-L1) [1]. ICIs have broad clinical activity across many cancers, including early-stage breast cancer, which affects >2 million women annually and causes >600,000 deaths from recurrence. Pembrolizumab improves overall survival in early-stage triple-negative breast cancer when combined with chemotherapy [2], and ICIs show encouraging preliminary activity in hormone receptor-positive (HR+) and HER2+ subtypes [3–5]. However, ICIs increase financial burden and can cause serious or irreversible immune-related toxicities such as adrenal failure, hypothyroidism, pneumonitis, and myositis/myocarditis. In addition, many patients still recur despite the use of ICIs; for example, 13% of patients with stage II/III triple-negative breast cancer die within 5 years of surgery despite receiving pembrolizumab [2].

These issues underscore a concerning clinical scenario whereby patients with breast cancer receiving ICIs may experience added cost and toxicity without clear benefit. This highlights the urgent need for biomarkers of ICI responses to carefully select patients who would most benefit from treatment. One promising biomarker approach is for pathologists to visually categorize tumors by their degree of immune cell infiltration within the tumor microenvironment. Specifically, stromal tumor-infiltrating lymphocytes (TILs; white blood cells including T-cells, B-cells, and innate lymphoid cells) can be visualized and quantified within the stromal tissue surrounding cancer cell epithelial nests. To date, this has largely been done using hematoxylin and eosin (H&E)-stained microscopic images obtained routinely during the diagnostic workup of breast cancer. Using these images, pathologists visually estimate the mean proportion of stromal area infiltrated by TILs (0%–100%). This method is intuitive, inexpensive, and widely accessible. Increasing TILs % is prognostic for long-term cancer-free survival and may also predict chemotherapy response. Consensus scoring guidelines and training modules are publicly available from the International TILs Working Group [6].

However, several caveats limit visual H&E TILs scoring as a prediction biomarker for ICI responses. First, visual scoring has inter-observer variability, especially among less experienced pathologists and in tumors with spatial heterogeneity [7]. Second, a mean TIL value collapses spatial features (i.e., autocorrelation, dispersion/distribution, heterogeneity, and distance) into a single metric, therefore discarding information that may contribute to prediction accuracy. Third, concordance is strong for clearly “high-TIL” or “low-TIL” tumors, but borderline cases near classification thresholds are less concordant. Finally, scoring of TILs by routine H&E cannot differentiate between immune cell subtypes (i.e., effector T cells, regulatory T cells, B cells, or natural killer cells) even though these cellular subtypes may have distinct prognostic significance [8].

**Fig 1:**
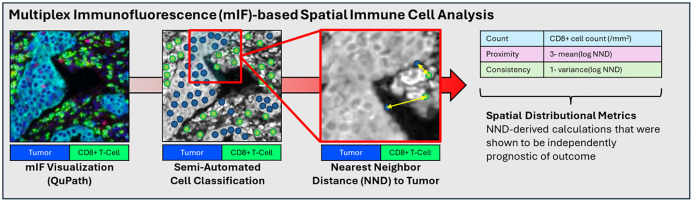
Profiling tumor-infiltrating lymphocytes (TILs) scores using multiplex immunofluorescence (mIF). Images show sequential representations of a multiplex immunofluorescence (mIF)-based spatial immune cell analysis. Multiplexed whole-slide images are digitized and subjected to a semi-automatic cell classifier using QuPath software. Subsequently, the nearest neighbor distance (NND) from the CD8+ T-cell to tumor is calculated and converted to metrics including count, proximity, and consistency, which have prognostic implications. Images adapted from [9].

In a recent *PLOS Medicine* study [9], Walker and colleagues show how spatial profiling of TILs using a next-generation microscopy approach (multispectral immunofluorescence, mIF) with digital image analysis (QuPath) has the potential to address some of these limitations by generating a TILs score with improved prediction/prognosis accuracy. mIF uses multiple fluorescence-conjugated antibody stains to characterize immune cells by phenotypic subtype, while QuPath is an open-source software that uses supervised machine learning to identify immune cell subtypes and their geographic coordinates from digitized microscopy images (see Fig 1) [10]. Using microarray cores from 1,467 participants in the Carolina Breast Cancer Study, the investigators characterized the spatial distributions of tumor cells in relation to CD8+ effector T cells, a tumor-killing subtype that has established prognostic relevance across multiple cancer types. This approach has potential to reduce errors related to human subjectivity and visual averaging, and allows for calculation of spatial metrics that describe how immune cells are organized in relation to tumor cells, rather than just the immune cell density.

The authors made several important observations. They demonstrated many HR+ tumors exhibited high CD8+ counts and tumor cell proximity, challenging the paradigm that HR+ tumors are less immune sensitive. Moreover, spatial characteristics beyond CD8+ density, such as the proximity of CD8+ cells to tumor cells and the consistency of CD8+/tumor interactions across sampled regions, were prognostic in both HR+ and HR− tumors (Fig 1). This was true even after adjusting for CD8 density and clinical covariates. These metrics correlated well with whole-slide spatial TIL features such as intratumoral and peritumoral strength derived from H&E digital image analysis, suggesting that smaller microarray-based measurements can capture broader tumor-immune architecture [10]. This work also provides elegant proof-of-concept that spatial configurations of TILs hold prognostic information beyond the mean immune cell density. It suggests a potential strategy to address one of the main limitations of visual TIL assessment: misclassification of tumors with intermediate TIL levels [7]. Analogous to HER2 testing, where an orthogonal assay (fluorescence in situ hybridization) resolves equivocal IHC 2+  results, spatial metrics such as proximity or consistency could serve as features to refine TILs classification and improve robustness near decision thresholds.

Together, this study [9] provides compelling preliminary evidence of the clinical utility of spatial TILs profiling; however, formidable work lies ahead before the approach can be adopted in the clinic. The mIF-based approach is resource-intensive and requires specialized infrastructure, and therefore, it would be important to demonstrate a substantial improvement in prediction/prognostic utility beyond what is afforded by classical H&E visual TILs scoring. It would also be important to show that mIF-based analysis adds value compared to a more economical alternative of digital H&E-based analysis using QuPath or fully automated digital AI analyses. Furthermore, the reported spatial analyses of Walker and colleagues relied largely on variations of a single proximity measure: the nearest-neighbor distance between CD8+ and tumor cells. However, a wide array of spatial metrics exist in the literature [11], adopted from fields of ecology, geography, and computational pathology, each having unique characteristics that may be better suited to characterize tumors. Methods such as Ripley’s K and related functions, mark-correlation analyses, or graph-based models of tumor–immune interactions may capture complex spatial behaviors, adjust for sampling effects/tumor architecture, and therefore may enhance prognostic and prediction accuracy. These alternative approaches should be compared to metrics evaluated in this study using the Carolina Breast Cancer Study and other spatial datasets.

The greatest opportunity to demonstrate clinical utility of spatial TILs profiling lies in the prediction of ICI benefit in the context of randomized phase III ICI clinical trials. For instance, the ongoing phase III NCTN trial SWOG S2206 (NCT06058377) is testing neoadjuvant chemotherapy ± durvalumab (anti-PD-L1 ICI) in high-risk HR+ stage II–III breast cancer (*n* = 3,680). Here, surgical tumor response (pathologic complete response, pCR) and long-term event-free survival (EFS) are being used as validated clinical outcomes. Baseline biopsies could be used to assess whether spatial TILs analyses (H&E-based or mIF-based) could improve upon prediction of pCR/EFS outcomes and ICI benefit, relative to conventional H&E visual TILs scoring. Spatial metrics could also be tested as orthogonal features to reclassify TIL-intermediate tumors at highest risk of visual misclassification. Similarly, the SCARLET trial (NCT05929768; SWOG S2212), enrolling HR– patients randomized to standard versus de-intensified chemotherapy + pembrolizumab (anti-PD1 ICI, *n* = 2,400), provides an opportunity to evaluate whether TIL-based biomarkers can guide chemotherapy de-escalation. Evaluating spatial TILs metrics in conjunction with broadly adopted clinical assays such as genomic risk scores (e.g., MammaPrint and Oncotype Dx) and PD-L1 testing would be an essential step before clinical adoption. Open access and collaborative frameworks will also be critical to allow for external validation, discovery of novel spatial metrics, and training the next generation of investigators in the emerging field of spatial TILs analysis.

Traditional prognostic biomarkers such as PD-L1 immunohistochemistry and visual TILs are limited by subjectivity, heterogeneity, and modest predictive performance. Digitization, machine learning, and spatial analytics offer a promising path forward to addressing these limitations. The work of Walker and colleagues [9] demonstrates that spatial features of immune infiltration, particularly the proximity of effector CD8+ cells to tumor, may outperform traditional density measures. However, their work underscores the need for more comprehensive exploration and validation of high-dimensional spatial data afforded by digital histology and machine learning.

## Abbreviations

EFS: event-free survival
ICIs: immune checkpoint inhibitors
mIF: multispectral immunofluorescence
NND: nearest neighbor distance
pCR: pathologic complete response
TILs: tumor-infiltrating lymphocytes
